# Environmental hazards of wastewater disposal on groundwater at the West Sohag site, Egypt

**DOI:** 10.1038/s41598-025-21565-7

**Published:** 2025-10-17

**Authors:** Shaymaa Rizk, Mariam Anis, Merna Nazer, Ahmed M. Youssef, Mostafa Redwan, Bosy A. El-Haddad

**Affiliations:** Geology Department, Faculty of Science, Sohag University, Sohag, 82524 Egypt

**Keywords:** Heavy metals, Environmental hazards, Land use, Geochemical investigation, Environmental sciences, Hydrology

## Abstract

Effluent infiltration from wastewater treatment plants into groundwater systems can be a source of contaminants of emerging concern that are not fully removed during the treatment processes. In the lowland desert area in Upper Egypt between the Eocene Limestone plateau and the new floodplain, wastewater disposal plants have been set up. Woody farmland will be irrigated with the treated wastewater. Some wastewater disposal sites, including the west Sohag site, have been operating since 1990. This site is considered a very hazardous source of soil and groundwater pollution. The current work aims to assess the environmental impact of the sewage water treatment plants (west Sohag site) in the Sohag Governorate, Egypt, using remote sensing and geochemical techniques. Monitoring the continuous extent of sewage water leakage and heavy metal mobility in the groundwater. The detailed visual interpretation of the different remote sensing data showed that the contaminated areas increased substantially. The area was classified into four classes, namely Urban centers, agricultural areas, water courses, and barren lands. The study showed that insufficient land is available to accommodate the projected quantities of wastewater. The hydraulic conductivity varies between 0.29 and 3.72 m/day with an average porosity of 38.9%. Excess raw wastewater accumulates on the ground surface at current operating sites, forming large, uncontrolled ponds. When soil, crops, and water supplies are contaminated chemically and bacteriologically, such ponds pose a risk to the ecosystem and could have catastrophic health consequences. Average heavy metal concentrations in the analyzed groundwater decreased from Zn > Cu > Pb > Cd, with mean values of 8.55 > 0.421 > 0.282 > 0.207 ppm, respectively. The correlations between heavy elements in the water are very high, with correlation coefficients greater than 0.8 between Pb & Cd, Pb & Cu, Pb & Zn, Cd & Cu, Cd & Zn, and Cu & Zn. According to speciation analysis, Zn, Cu, Pb, and Cd are highly mobile metals in the study area. Our findings confirmed the occurrence of sewage water leaking into the groundwater aquifer in the study area. These results offer useful data for examining the transport properties of heavy metal contaminants and developing practical remediation strategies.

## Introduction

Wastewater management is a serious challenge in metropolitan regions of developing nations, as fast population expansion, industrialization, and urbanization pressure existing infrastructure^1,2^. Numerous municipalities in developing countries have inadequate sewage infrastructure, discharging wastewater into rivers, lakes, and coastal areas^3^. Accordingly, ineffective management of wastewater can lead to the contamination of water bodies, which poses significant risks to public health and the environment^4,5^. Significant risks to the environment and human health are associated with wastewater disposal in an open area^6^. It makes it easier for waterborne diseases like cholera, dysentery, and typhoid to spread in urban areas^7^. Several authors^8–10^ provide an analysis of land use that influences groundwater quality. Environmental dangers have recently become one of the most serious worries for most governments worldwide due to the adverse effects of improper wastewater disposal^11^. The majority of the risks associated with heavy metals, nitrates, salts, microbes, and poisonous organic and inorganic compounds are associated with the presence of wastewater^12^. Heavy metal contamination in aquatic environments has attracted global attention because of its abundance, persistence, and environmental toxicity^13^. Heavy metals are abundant in the environment due to both natural and man-made activity, display hazardous effects, and severe health problems at lower concentrations^14–16^. The United States Environmental Protection Agency (US EPA) and the International Agency for Research on Cancer (IARC) categorized heavy metals as human carcinogens.

The global population is projected to reach eight billion by 2025 and 9.3 billion by 2050, with approximately 70% of that population residing in urban areas^17,18^. Cities worldwide will face a formidable challenge in waste management due to the continued growth of the economy, the enhancement of lifestyles, and the rise of consumerism. Groundwater is the key source of drinkable water in several metropolitan areas. The degradation of the environment leads to groundwater contamination^19^. Making investments in wastewater treatment infrastructure, which may include sewage treatment facilities, can make it possible to facilitate the appropriate processing of wastewater before its discharge into the environment^20,21^. These strategies can also reduce dangers and improve the quality of life in metropolitan areas.

One of the most densely populated regions of Egypt is Sohag Governorate. The conventional wastewater disposal technique in this Governorate’s inhabited areas makes use of unsuitable residential cesspools. In both rural and urban areas, it has resulted in severe environmental and health issues^22^. To properly dispose of such highly-risk wastewater, public sewage systems and treatment facilities must be established immediately. Currently, the only method for disposing of wastewater throughout the Nile Valley in Upper Egypt is land application. Thus, the treated effluent will be used to irrigate woodland farmlands in the lowland desert zone between the higher elevation Eocene limestone plateau and the farmed floodplain. The west Sohag disposal site has been chosen for this intend. It is located along the low desert zone of the old floodplain of the River Nile. This region’s desert district is small and situated in close proximity to essential infrastructure and service components, including residential neighborhoods, reclaimed lands, agricultural floodplains, and surface water supplies (the River Nile and related canals). Furthermore, the area’s Quaternary aquifer depends critically on the subsurface water-bearing sediments, which are primarily sand and gravel. This desert region is undergoing a number of development initiatives, such as reclamation, urbanization, and industry; notably, the Quaternary aquifer serves as the primary source of water for these projects.

The integration of remote sensing and groundwater quality explains the impact of cultivation, urbanization, and other human endeavors on the quality of groundwater^9^. Therefore, establishing wastewater disposal areas in this zone must be cautiously implemented. The recent integration of geochemical analysis with remote sensing and geographic information systems has received more attention in mapping contaminated areas in surface soil and groundwater^23^. These methods have advantages over traditional field surveys as they are cost-effective, especially in mapping contaminated sites. Remote sensing facilitates monitoring the effect of wastewater treatment sites on the ecosystem in large areas^24^. Various remote sensing data are essential in monitoring environmental hazards^25^.

As more locations for wastewater disposal are being suggested, it is essential to resolve the existing issues to prevent negative effects on the entire valley. Because it would be difficult to remediate such extensive, catastrophic environmental contamination in the future. It is necessary to evaluate the adverse effects that could affect various environmental elements. The objective of the current study is to apply remote-sensing techniques and geochemical studies to understand the environmental impacts of the wastewater disposal site on the groundwater aquifer system in the area. Additionally, geochemical analysis of heavy metals can be used to understand their distributions and impacts on the groundwater system. These heavy metals include Zn, Cd, Pb, and Cu.

This study will cover feedback on groundwater quality, degradation agents in this area, and the necessity of appropriate groundwater management and monitoring for long-term sustainability. Human health and the long-term viability of civilization are adversely affected by the decline in drinking water quality. Improper wastewater disposal exacerbated this problem in many areas, leading to increased groundwater pollution. The novelty of this work lies in evaluating the combination of multivariate statistical methods, remote sensing, and geochemical parameters to study groundwater contamination and detect polluted areas around a sewage wastewater disposal site using high-resolution rectified Google Earth Pro images. These techniques are helpful for determining the geochemical processes governing groundwater evolution and for examining the quality of groundwater.

### Study area and its geological setting

**Fig. 1:**
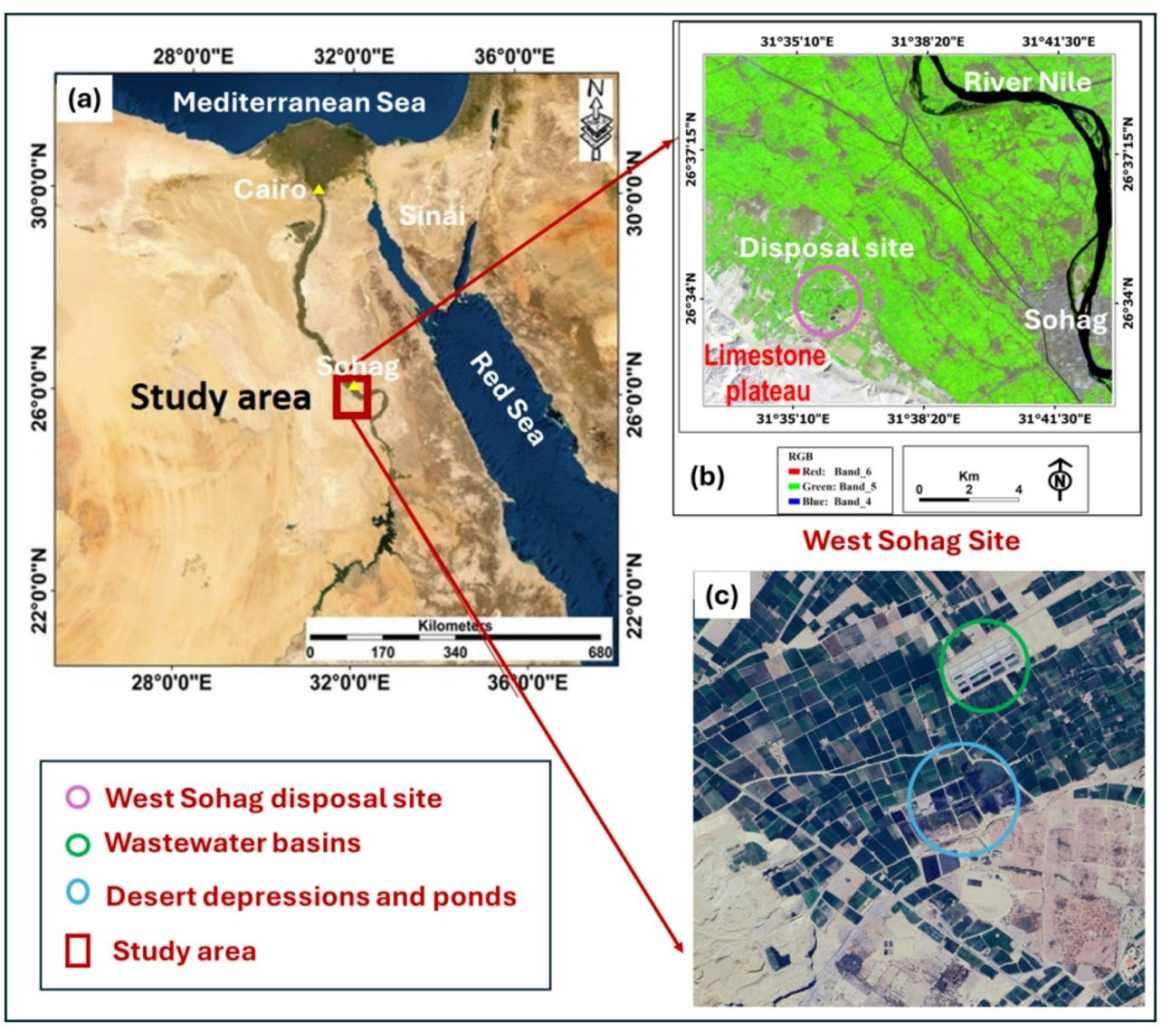
**a** Location map of the study area in Egypt; **b** west Sohag disposal site with the surrounding agriculture and urban areas till the river Nile (Landsat 15-m image); **c** the wastewater basins and ponds (high resolution Google Earth image) (the maps were prepared by the authors using ArcGIS 10.8 (https://www.arcgis.com)).

The study area represents a portion of the Nile Valley, stretching between latitudes 26° 10′ 00″ and 26° 35′ 00″ N and between longitudes 31° 15′ 00″ and 32° 00′ 00″ E (Fig. 1). Within this area, the location of the west Sohag disposal site and its surrounding areas, extending east to the Nile River, are shown on the west side of the River Nile in Fig. 1b. Additionally, high-resolution images showing the disposal site along the low desert are presented in Fig. 1d.

**Fig. 2:**
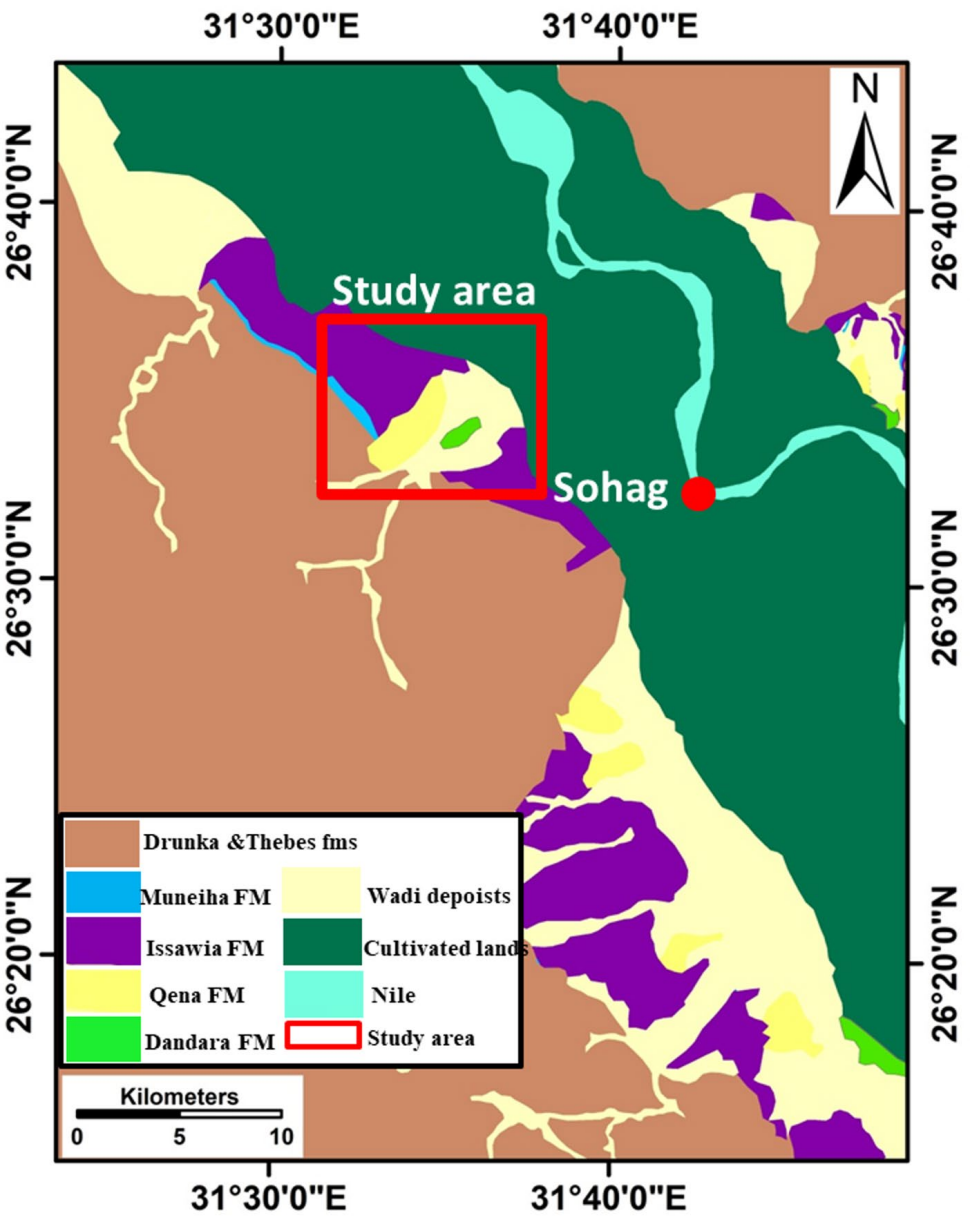
Geological map of the study area showing the various rock units and their areal distributions (Modified after Youssef et al.^18^).

The Nile Valley geology has been comprehensively studied by various authors^26–30^. The Nile Valley is occupied by a buried canyon that was laterally filled with several types of clastics, which accumulated during the successive stages of the Nile’s evolution from its beginning (from the Lower Eocene to recent)^27^ (Fig. 2). These deposits come from a variety of sources and form under a range of climatic and depositional circumstances^30^. These rock units can be categorized into four groups: (1) The Eocene deposits represented by Thebes and Drunka formations. Thebes is a massive, layered limestone with flint bands or nodules and marl-rich strata with Nummulites and planktonic foraminifera. Drunka is a medium to thick-bedded succession of limestone, highly bioturbated in some horizons, and siliceous concretions of different sizes. (2) The Pliocene rock unit comprising the Muneiha Formation. It consists of fluviatile-dominated sediments of sand, silt, and mud intercalations as well as bedded clay intercalated with thin beds and lenses of silt and fine sand. (3) Pleistocene deposits, which comprise three rock formations, including Issawia, Qena, and Dandara formations. Issawia “Early Pleistocene” is composed of two facies, carbonate in the core zones and clastic near the lake’s edges. Qena “Middle Pleistocene” is consist of quartzose sands and gravels lacking igneous and metamorphic fragments^27^. Dandara “Late Pleistocene” is a fluviatile fine sand-silt intercalation, accumulated in low-energy environments. (4) The recent or the Holocene sediments, which are represented by the alluvial and wadi deposits. Alluvial deposits are clays and silts with sandstone intercalations. Whereas wadi deposits consist of sand and gravel disintegrated from the Eocene limestone plateaus and reworked material from old sediments.

The limestone plateau that borders the study area on the east and west is divided by numerous wadis that run primarily in the E-W directions. Low desert land areas follow these plateaus inward, followed by the agricultural areas along the River Nile. Three major units are among the primary geomorphological elements in the studied area (Fig. 2), and each unit has its characteristic shape, pattern, and relief. Thses units include the Eocene limestone plateau, the low-land desert area, and the Nile floodplain (Geomorphology section). 

### Hydrogeological setting

The Quaternary aquifer is composed of Pliocene clay, which serves as the base of the aquifer. Pleistocene fluviatile sediments are covering it in the cultivated lands. These Pleistocene sediments are covered by the floodplain sediments. The thickness of the aquifer varies from 20 to 80 m and increases towards the east. The hydraulic conductivity varies between 0.29 and 3.72 m/day. In Sohag, the aquifer system is recharged by the infiltration of surplus irrigation water that leaks vertically beneath cultivated lands and seeps horizontally towards reclaimed desert lands through seepage from irrigation canals, such as the west Sohag area. These canals play a crucial role in shaping the water table pattern of the aquifer and obtain their water from the dominant Nile water. It is also important to note that these canals play a crucial role in recharging the surrounding aquifer.

## Data used

### Remote sensing data

This study used remote sensing techniques to monitor the wastewater contamination in the west Sohag site. Various remote sensing data were used in this study, including the ALOS digital elevation model (DEM-30 m), Landsat-8, and Google Earth Pro images (Table 1). A cloud-free Landsat-8 OLI image was obtained on January 24, 2025. The image projection is Universal Transverse Mercator zone 36 N and the WGS-84 datum. We used scene Path175–Raw42 to prepare a multispectral image for each study area’s land use change analysis. Multispectral Landsat images with 15-m spatial resolution were generated using eight bands (1, 2, 3, 4, 5, 6, 7, and 9) and a resolution merge technique with the help of a panchromatic band (15-m spatial resolution). Google Earth time series images were used to understand the distribution of wastewater ponds with time (data from 1990 to 2025).

**Table 1:** Remote sensing data characteristics.

Data type	Acquired time	Bands	Final resolution
Landsat OLI	2025	1, 2, 3, 4, 5, 6, 7, 9 (30 m) and 8 (15 m)	15 m
Alos DEM	2020	One band 30 m	15 m
Google Earth Pro.	1990–2025	Three bands < 0.5 m	< 0.5 m

### Fieldwork and sampling

**Fig. 3:**
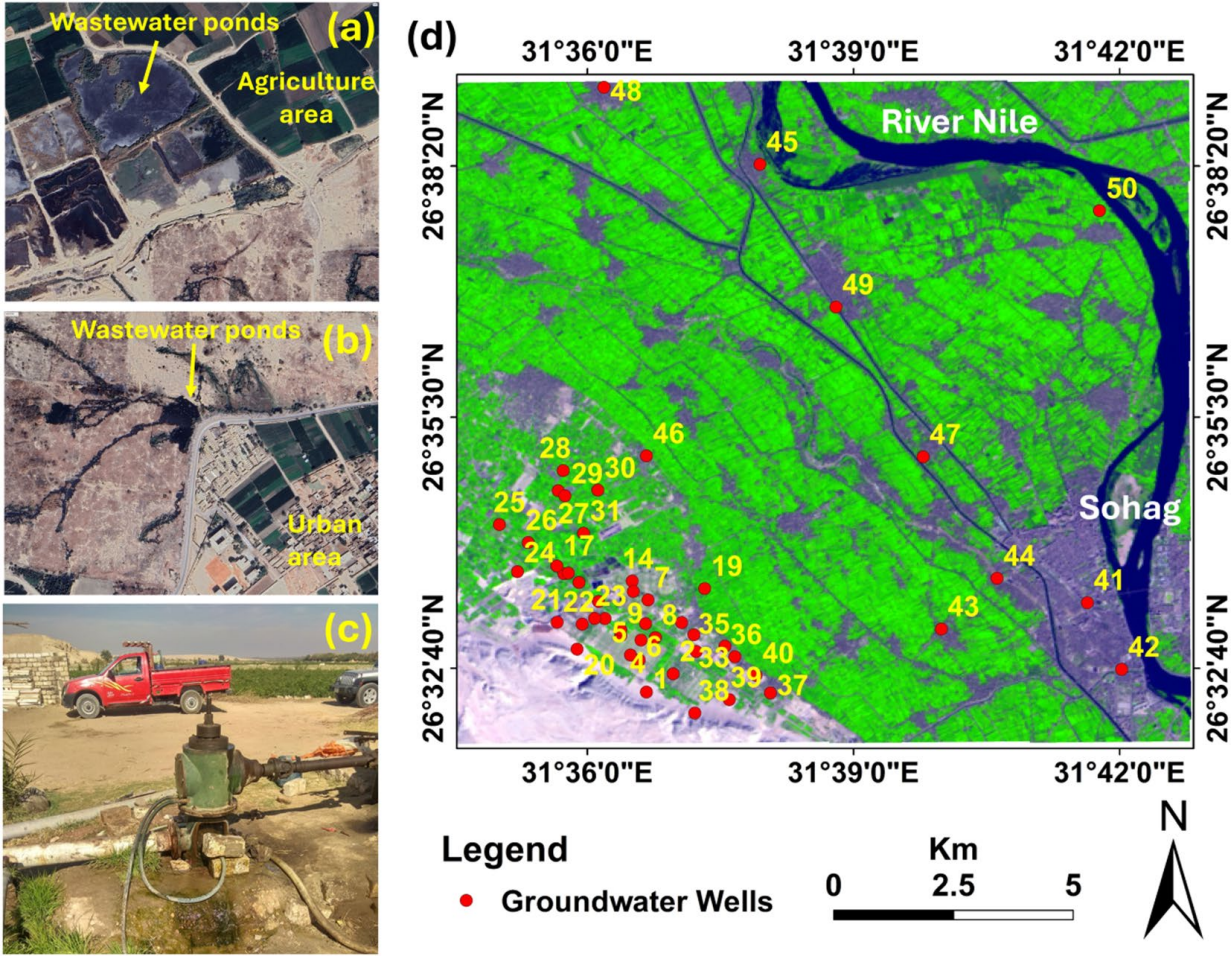
**a** Wastewater ponds surrounded by agricultural areas (extracted from Google Earth image May, 2025), **b** Wastewater ponds to the west of urban areas (extracted from Google Earth image May, 2025), **c** Private farmers irrigate their farms with Groundwater well which used to collect samples (photo by the authors), and **d** distribution of water wells from which water samples were obtained from the west Sohag disposal site area and its surroundings (Map prepared by the authors using ArcGIS 10.8 (https://www.arcgis.com)).

West Sohag wastewater disposal site has already been operated. Currently, effluent will be disposed of and used to irrigate woody farmlands. There is not enough land area at the west Sohag site to handle the amount of wastewater that needs to be disposed of, according to an initial analysis of the anticipated wastewater volumes and the woody land areas designated for irrigation. Large, uncontrolled ponds are created at the operational site when excess wastewater builds up on the ground surface^18^. These wastewater ponds pose serious health dangers and are important sources of environmental contamination (Fig. 3a). These wastewater ponds are not surrounded by buffer zones; they are surrounded by agricultural areas of the reclaimed regions, cultivated floodplains of the River Nile, Urban areas, and critical utilities (irrigated channels and groundwater aquifer system). Wastewater has even been utilized illegally by private farmers to irrigate their land (Fig. 3b). In addition, farmers dig groundwater wells very close to the disposal sites and ponds and used the water for irrigation and domestic use (Fig. 3c). The close proximity of wastewater disposal sites to populated areas and the River Nile also presents other challenges; if the soil levees enclosing and containing wastewater ponds fail, major problems could result (Fig. 3d). The main goal of any wastewater application on land is to prevent contamination of the soil and groundwater. At these locations, wastewater leakage along the surface and downward to the groundwater aquifer poses a serious risk. Wastewater at these locations may become contaminated in two ways: chemically by organic and inorganic components, and bacterially and virally.

To start an inquiry into this issue, a methodical approach was taken to gather field data and examine the groundwater’s geochemical properties. Quaternary groundwater samples were collected from 50 private wells (farm wells) dispersed around the west Sohag site (Fig. 3d). The samples were collected and stored in polyethylene bottles of 1-L capacity and preserved at 4 °C until examination. The bottles were washed numerous times with deionized water, followed by four times with the groundwater to minimize the chance of any pollution from the surrounding. The current study used the sampling technique outlined by^31^.

## Methodology

**Fig. 4:**
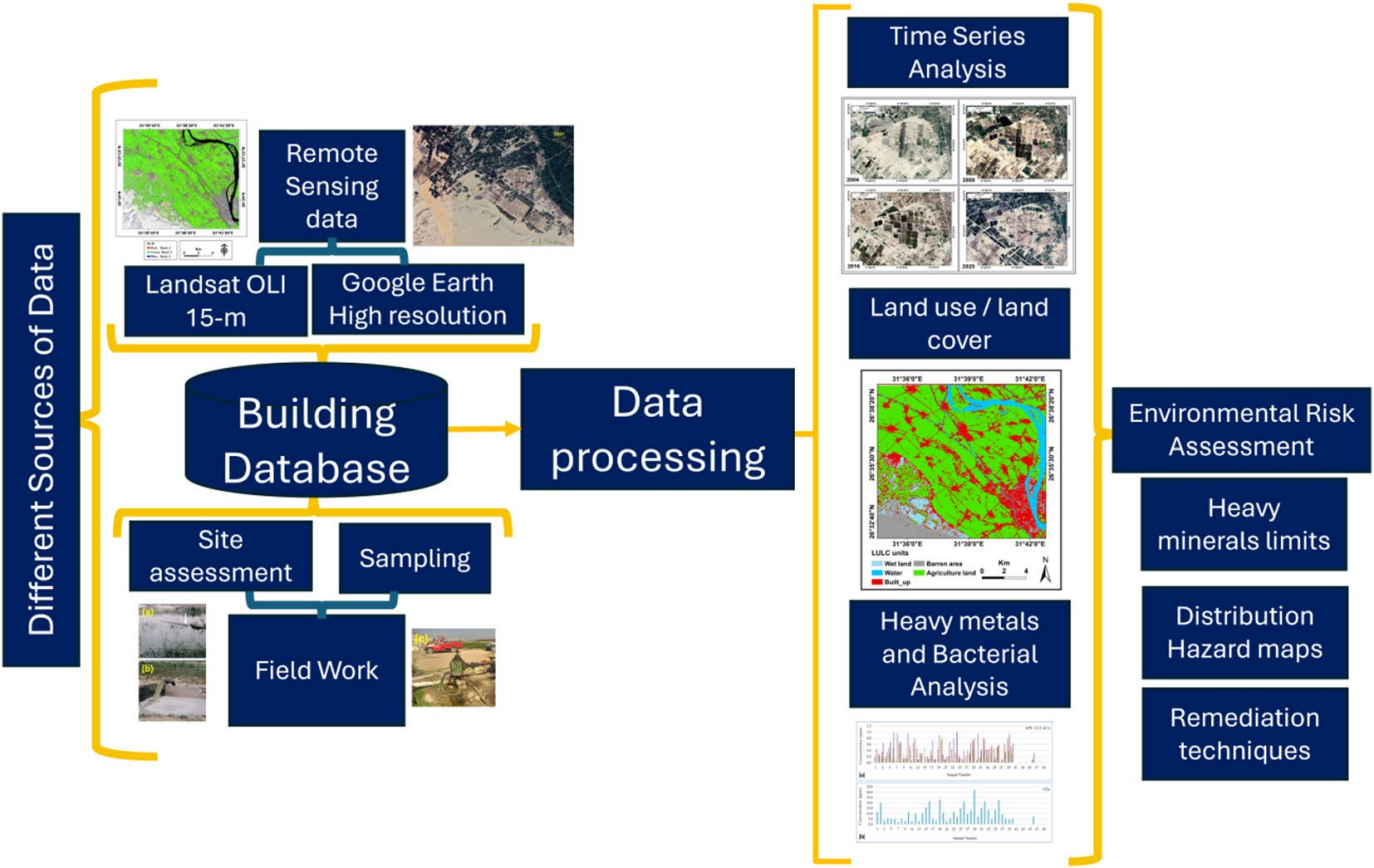
Flow chart showing the methodologies used in the current study.

Different methodologies were utilized to study the impact of the wastewater disposal site on the groundwater aquifer system in the area and its surrounding agricultural activities, and the critical water elements in the area. Figure 4 is the flow chart describing the study phases from collecting the data and sample, preprocessing and processing the data, chemical analysis, interpretation, and finally recommendation to minimize the effect of the contaminated materials on the soil and water in the area and its surroundings.

### Remote sensing data analysis

Many studies have proven the ability of remote-sensing techniques to detect and map surface area contamination^32,33^. DEM was used for the study area using ALOS with a 30-m spatial resolution to understand the geomorphological and topographic variations and extract the geomorphic parameters (slope gradients, elevation, and cross sections). A Landsat-OLI image with a 15 m resolution was used to prepare the study area’s land use map. Google Earth Pro images were used to understand the dynamic changes in wastewater disposal sites and their surroundings over time.

### Laboratory and statistical investigation

First, the total heavy metal concentrations were determined for the 50 groundwater samples, as shown in Fig. 3d. These samples were collected during two field trips on 5 and 10 January 2022.

#### Water samples preparation for AAS analysis

Groundwater should be filtered to avoid any particles that will damage the atomizer. Additionally, dilute water samples and acidify groundwater samples with HNO_3_ (AR) to prevent metal precipitation. Concentrations of the investigated metals (Zn, Cd, Pb, and Cu) were measured in the water samples utilizing an atomic absorption spectrophotometer (AAS) (Perkin Elmer, Analyst 400) according to the following table (Table 2).

**Table 2:** Standard conditions and characteristic concentration check for atomic absorption.

Element	WL (nm)	SBW (nm) flame gases	Char conc. check
Zn	213.9	0.7	1.0
Pd	244.8	0.2	10.0
Cd	228.8	0.7	1.5
Cu	324.8	0.7	4.0

Additionally, bacteriological analysis has been conducted to assess the impact of wastewater contamination on the groundwater aquifer. Because it is impossible to test water for every type of disease-causing organism, states typically look to identify bacterial indicators^34^. The bacterial indicator indicates that a certain selection of water may be contaminated with untreated sewage and that other, more hazardous organisms are also present. The MacConkey broth media and multiple tube fermentation procedures were used to perform the bacteriological examination. After 24 h of incubation at 44.5 °C, the fecal coliform was identified. The bacteriological analysis was carried out in accordance with the guidelines of the APHA^35^.

Groundwater analysis was conducted in duplicate, and high-purity-grade chemicals were used to create suitable standards and blanks, ensuring maximum precision in measurements and quality control. Procedure blanks and quality control samples prepared from standard solutions were used to ensure the accuracy of the samples. The quality control of the reported results was demonstrated using certified reference materials (CRMs), blank reagents, and duplicate samples.

The descriptive statistics include the mean, standard deviation, minimum, maximum, and median. Correlation and advanced statistical analysis of the different heavy metals were performed using Statistica 13.6.0, StatSoft Inc., and JMP software 17.0 to interpret the relationships between heavy metals and estimate the significant sources of heavy metals in soil samples. In addition, based on the contamination indices, the risk assessment of the heavy metals was calculated to understand their impact on health and domestic use.

### Comparison analysis and environmental hazard mapping using GIS

The samples from the wastewater disposal site and the Nile Valley floodplain were compared using heavy metal values, statistical analysis of the heavy metals, and advanced statistical techniques. Final distribution maps showing the heavy metal distribution in the study area were prepared to outline the most polluted areas.

## Results

### Permeability and textural relationships

**Fig. 5:**
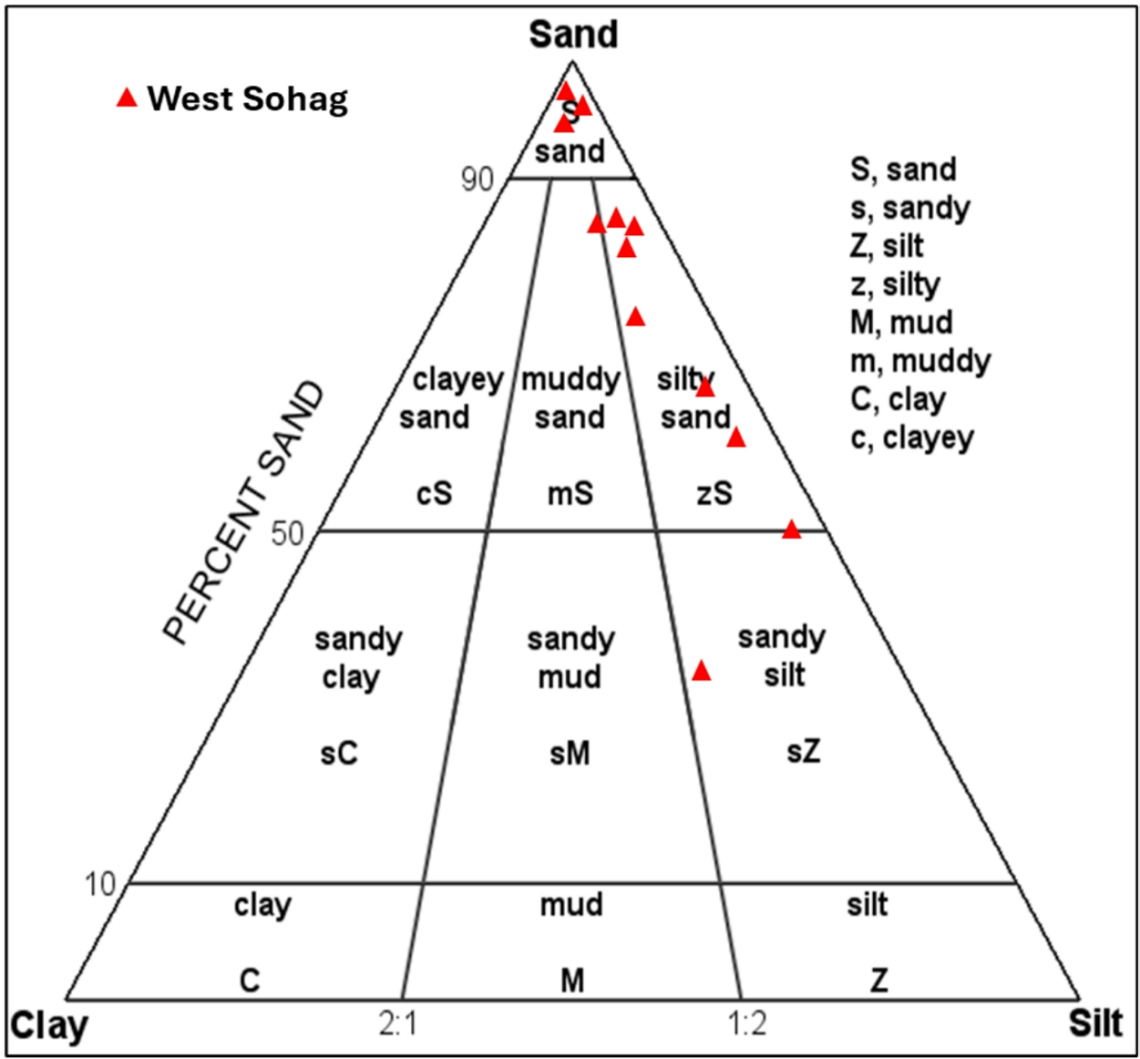
The texture characteristics for the wastewater disposal site.

Under the right circumstances, the accumulated wastewater can seep into the soil and be utilized by plants to thrive. The effluent might, however, also quickly seep downward and make its way to the groundwater aquifer system. The sediments’ textural properties, which influence their permeability and the wastewater migration rate, regulate the matter. The compaction and textural properties of sediments are the primary determinants of hydraulic conductivity (Table 3)^36,37^. Sediments with hydraulic conductivity less than 0.03 m/day are classified as very low conductive; sediments with hydraulic conductivity in the range from 0.03 to 0.12 m/day are classified as low conductive; sediments with moderate conductivity have values ranging from 0.12 to 3 m/day; and sediments with hydraulic conductivity ranging from 3.0 to more than 6 m/day are characterized as highly conductive. The problem was quantified by measuring the textural properties of the sediments at the west Sohag disposal site and empirically estimating the hydraulic conductivity, which is a property of sediment to transfer water. It is observed that most of the sediment samples from the west Sohag site were grouped into muddy sand, silty sand, sand, and sand-gravel (Fig. 5). The hydraulic conductivity varies between 0.29 and 3.72 m/day, with an average porosity of 38.9%. The hydraulic conductivity of the soil at the site reflects moderate to high permeability. We would anticipate that the wastewater would quickly move downward and reach the groundwater since the sediments are so conductive. It is anticipated that a similar situation will arise at the many suggested wastewater disposal locations along the Nile Valley, most likely leading to severe health and environmental issues for individuals who use the groundwater supplies.

**Table 3:** Hydraulic conductivity (K) estimates (mean values) for various sediments.

Sediment type	Standard code	Hydraulic conductivity
Gravel	A	1000 m/day
Sand	B	10 m/day
Silty sand	C	1 m/day
Silt	D	0.1 m/day
Fracture till, clay, or shale	E, F	0.001–0.0001 m/day
Massive till or mixed sand-silt-clay	G	0.00001 m/day
Massive clay or shale	H	0.000001 m/day

### Geomorphology and cross-section model for the study area

**Fig. 6:**
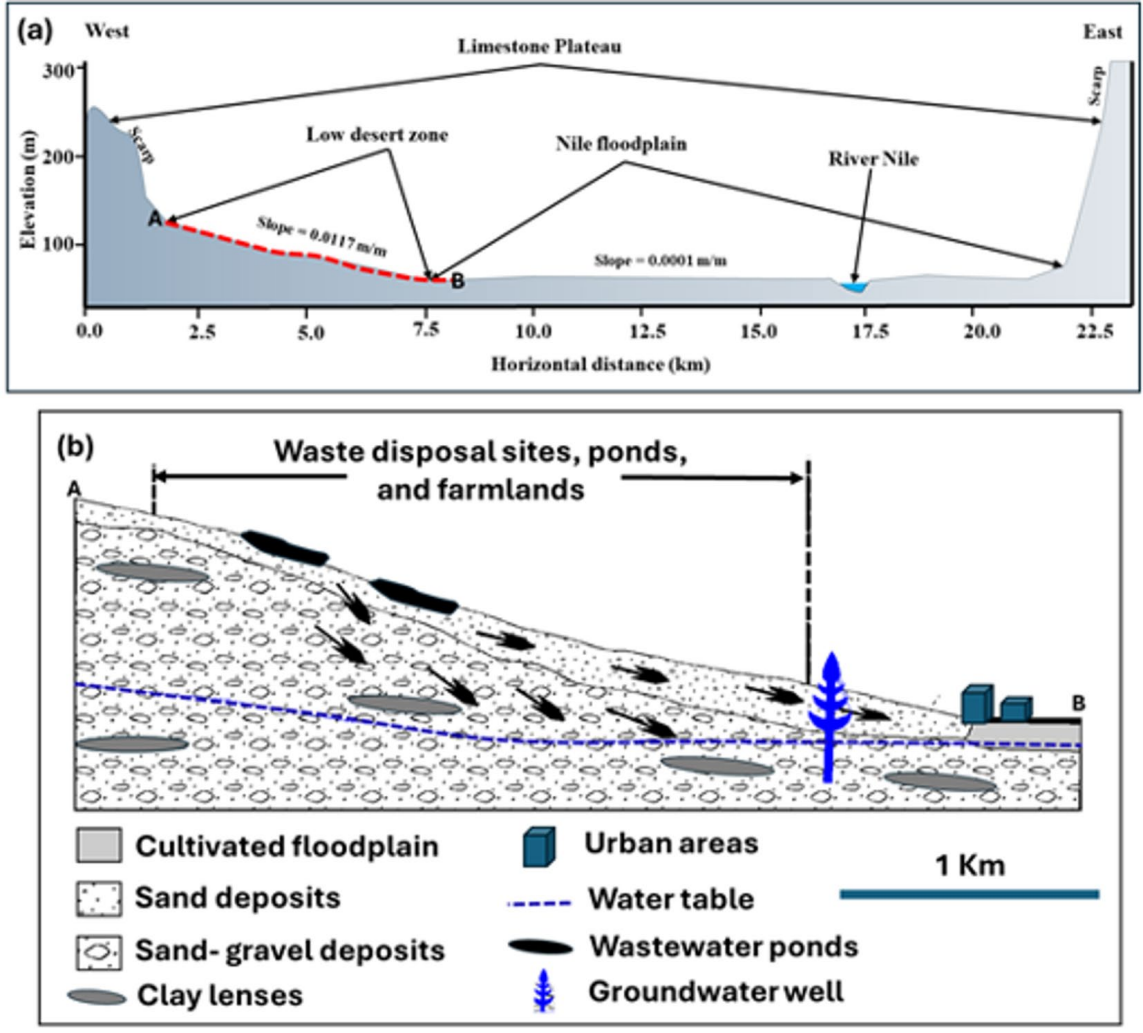
**a** The detailed cross-section from West to East along the west Sohag wastewater disposal site, **b** detailed cross-section (A-B) from West to East for the low desert zone and the first section of the flood plain (for west Sohag wastewater disposal site).

An exploratory field observation was conducted to gain familiarity with the area’s general conditions. Based on the location of the wastewater disposal facility, a cross-section from west to east was selected (Fig. 6a). Based on this cross-section that moves through the west Sohag wastewater site, it was found that the western part of the Sohag area has three main geomorphologic zones: the Eocene limestone plateau, the low-land desert area, and the Nile floodplain. (1) The Eocene limestone plateau is characterized by elevations ranging from 190 to 350 m and some geomorphologic features, such as limestone scarps, hanging valleys, and cavities. Many dry wadies that run toward the Nile Valley dissect the limestone plateau’s surface. Occasionally, these wadies receive large quantities of rainfall, forming flood hazards. (2) The second one is the low desert zone, which captured our interest due to the occurrence of the west Sohag site. It stretches between the Eocene limestone plateau and the farmed floodplain on both sides of the Nile Valley. These regions make up the ancient alluvial plains, which are symbolized by a sequence of progressively higher terraces that range in elevation from 70 to 190 m. This zone is characterized by the most reclaimed lands and the new development projects. Agriculture and urban activities are common in this zone. It is characterized by gentle to moderate slope topography with a slope gradient from 0.008 to 0.01 m/m along the low desert zone. (3) The third unit is the Nile floodplain, which comprises the adjacent, leveled cultivated lands spread out along the Nile River’s eastern and western banks. Fertile clay and silt sediments from farming activities make them up. The floodplain of the Nile dips gradually to the north and is nearly level. A nearly level surface is shown by the cross-section, particularly for the Nile floodplain zone, with a slope gradient of 0.0001 m/m from west towards the Nile River. Its elevation in Sohag varies between 55 and 70 m. It extends from the low desert zone to the Nile. In the study area, we used some well logs that were dug in close proximity to the site to determine the thickness of various subsurface layers and the depth of the groundwater level. The aquifer has been assumed to be homogeneous and illustrates the problem for policymakers. The cross-section data revealed that the west cross-section indicates the majority of materials in the area are composed of sand and gravel, which could quickly infiltrate the groundwater aquifer. Figure 6b presents the detailed cross-section model to understand the possible pollution of the surface soil and groundwater aquifer, which will likely occur in the area.

### Land use mapping

**Fig. 7:**
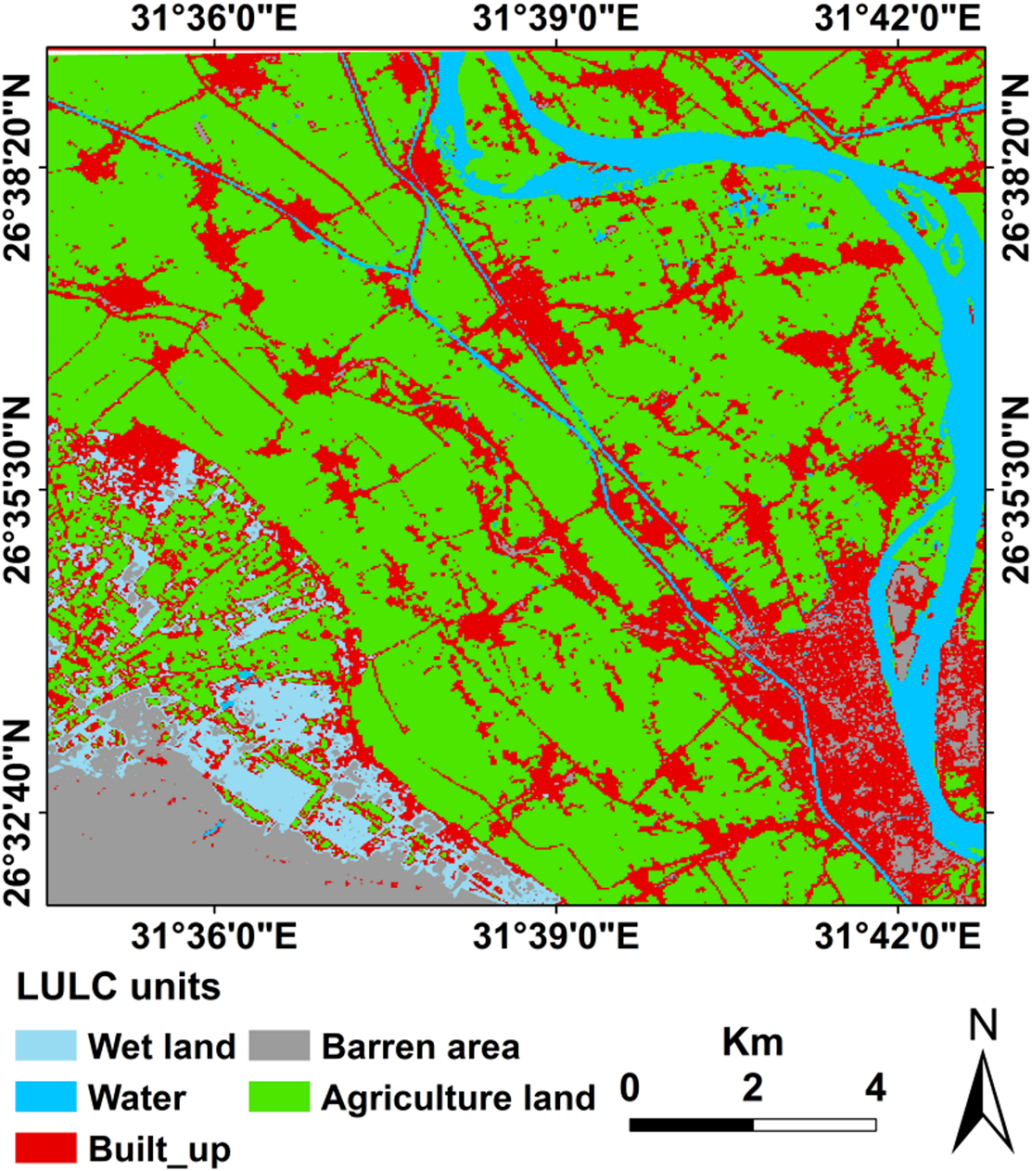
Detailed land use/land cover map for the study area (the LULC map was prepared by the authors using ArcGIS 10.8 (https://www.arcgis.com)).

From the detailed visual interpretations of the remote sensing data. It was indicated that agricultural activities are common practice in both areas. Broad areas are used for this purpose, including the cultivated initially lands in the floodplain, water course “channels and the River Nile,” and the newly reclaimed lands in the low desert zone (Fig. 7). This fact maximizes the importance of this research in understanding and monitoring the pollution of agricultural lands due to the sewage water treatment sites. The Landsat 8 OLI image of 2025 was used to detect and monitor land use/ land cover classes. In the current study, supervised classification using Machine Learning (Random Forest) was used to generate a land use map (Fig. 7). The area was classified into four main classes, including built-up areas, “Urban centers”, agricultural areas, water courses, and barren lands.

### Geochemical investigation

#### Statistical analysis and correlations

Data analysis was executed using SPSS version 26 (IBM, USA) and JMP version 17. The results of the descriptive statistics calculated for the four factors of 50 groundwater samples are given in Table 4. These variables include four heavy metals that are related to wastewater activities (Cu, Cd, Pb, and Zn). Skewness values are used to analyze the asymmetrical or symmetrical distribution of elements. Right skewed means a positive skewness when its value is above 0. However, left-skewed is negative skewness, where skewness is below 0, which means the distribution is not symmetrical.

**Table 4:** Brief descriptive statistics of various variables used in the current study.

Factors	Cu (ppm)	Cd (ppm)	Pb (ppm)	Zn (ppm)
Maximum	1.001	0.832	0.811	32.000
Minimum	0.008	0.000	0.002	0.012
Mean	0.421	0.207	0.282	8.550
Standard deviation	0.333	0.194	0.242	7.577
Skewness	0.306	1.154	0.586	1.027
Kurtosis	− 1.302	1.237	-0.788	0.662

The correlations between the variables were also ascertained using Pearson’s correlation coefficient. A P value of less than 0.05 was considered statistically significant in all analyses. Pearson and Spearman correlation indices were used to examine the relationships between these chemical variables. The results of the correlation between different variables are presented in Table 5. According to the results of this correlation, the correlations between these variables are classified as very high, with correlation coefficients greater than 0.8 between Pb & Cd, Pb & Cu, Pb & Zn, Cd & Cu, Cd & Zn, and Cu & Zn. Consistent with these findings^38^, in imposing the effectiveness of the distribution of heavy metals in land use sediments indicated that the correlation of the concentration of heavy metals in the water can be a cause for the efficacy of their distribution from one or more common causes (e.g., urban, industrial, agricultural activities and the underlying geological structure). Thus, the correctness of the results’ interpretations of applying different statistics in groundwater pollution investigations requires familiarity with local sources and situations that can potentially cause environmental pollution^39^.

**Table 5:** Multivariate correlations.

	Pb	Cd	Cu	Zn
Pb	1.000	0.914	0.896	0.819
Cd	0.914	1.000	0.882	0.865
Cu	0.896	0.882	1.000	0.851
Zn	0.819	0.865	0.851	1.000

### Heavy metals in the Quaternary groundwater aquifer in the West Sohag area

**Fig. 8:**
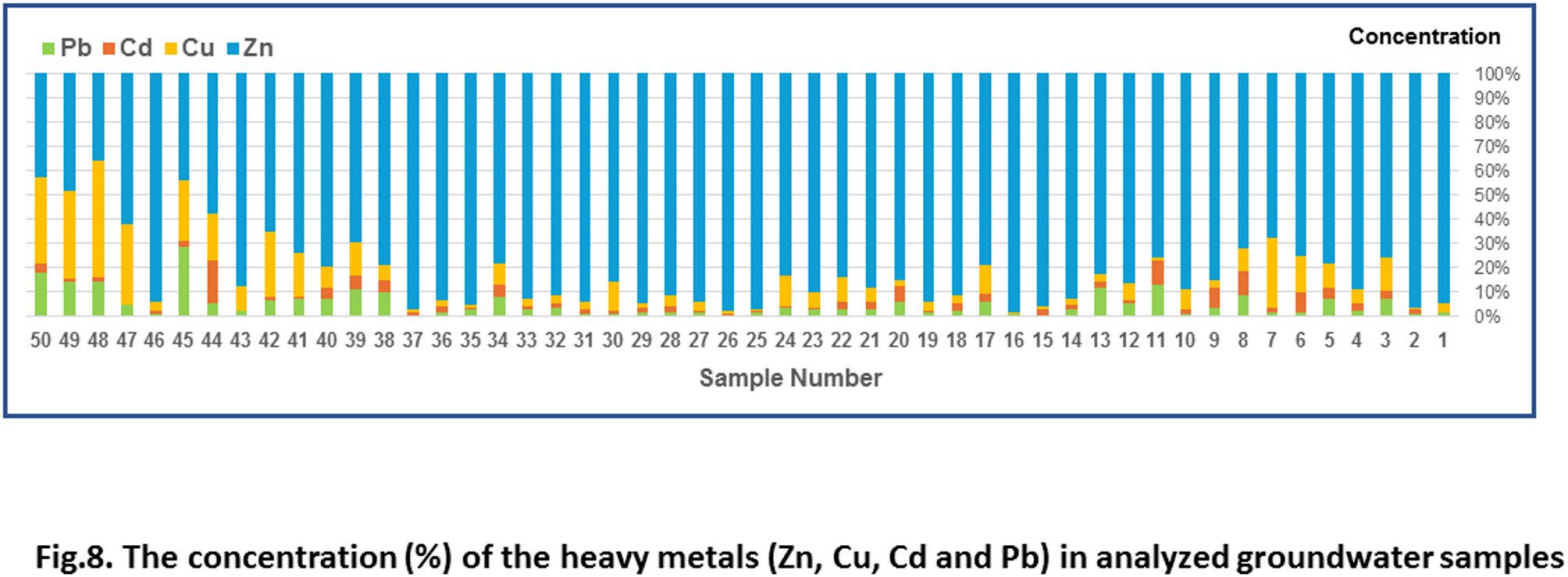
The concentration (%) of the heavy metals (Zn, Cu, Cd, and Pb) in analyzed groundwater samples.

The elevated levels of toxic heavy metals in west Sohag groundwater (Fig. 8) reflected the high concentrations of these heavy metals in Quaternary groundwater along the low desert zone, due to sewage water infiltration from wastewater ponds, tree farms, and wastewater basins. This indicates that the water is unsafe for domestic and agricultural use in the area. These contamination activities decrease towards the east (River Nile floodplain). However, over time, the slope of the area towards the River Nile could cause the polluted water with heavy metals to move towards the Floodplain aquifer system. In this case, a large number of people living in urban centers could be more vulnerable to these heavy metals and may experience long-term health issues. Average concentrations of heavy metals in the analyzed groundwater decreased from Zn > Cu > Pb > Cd, with mean values of 8.55 > 0.421 > 0.282 > 0.207 ppm, respectively.

### Distribution maps of various variables

Like a natural disaster, the contamination of groundwater aquifers by heavy metal hazards is challenging to manage due to several factors influencing it. The danger caused by heavy metals indicates the presence of wastewater ponds and excess use of fertilizers, which occur along the surface. A better knowledge of the amount of pollution is obtained by applying a variety of quantitative and qualitative groundwater pollution assessments and zoning techniques together. The values obtained from the chemical analysis of each factor were zoned using the inverse distance weight (IDW) method. The accuracy of the generated maps was adequate (RMSE = 0.007), (MAE = 0.006), and (MBE = 0.0005).

**Fig. 9:**
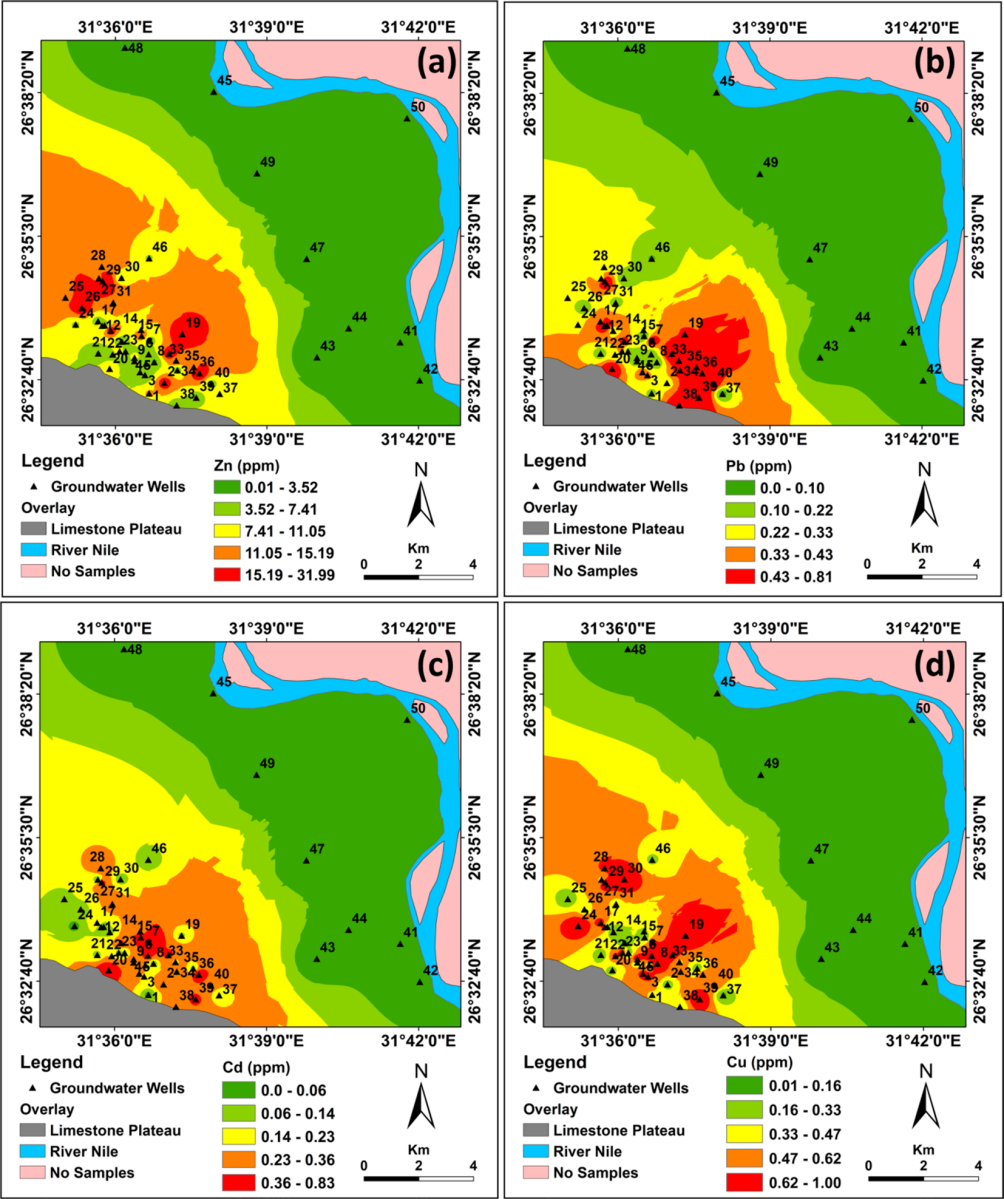
Distribution maps of the four important heavy metals in the groundwater aquifer west of Sohag area: **a** Zn, **b** Pb, **c** Cd, and **d** Cu (the maps were prepared by the authors using ArcGIS 10.8 (https://www.arcgis.com)).

Zn distribution map was generated as shown in Fig. 9a. Zn values ranged from 0.012 ppm (in the Nile floodplain aquifer) to 32.0 ppm (in the low desert zone aquifer near wastewater site), with an average value of 8.55 ppm and a standard deviation of 7.577 (Table 4). The Zn distribution was classified into five categories based on the natural break classifier: very high (> 15.19 ppm), high (11.05–15.19 ppm), moderate (7.41–11.05 ppm), low (3.52–7.41 ppm), and very low (< 3.52 ppm). The highest aquifer percentage in the low desert zone of the western part of the study area is characterized by moderate, high, and very high concentrations of Zn. In contrast, the low and very low concentrations of Zn are concentrated in the eastern side of the investigated area, specifically the Nile floodplain aquifer system.

Pb map distribution was generated as shown in Fig. 9b. Pb values range from 0.002 to 0.811 ppm, with a mean value of 0.282 ppm and a standard deviation of 0.242 (Table 4). It has been classified into five categories based on the natural break classifier: very high (> 0.43 ppm), high (0.33–0.43 ppm), moderate (0.22–0.33 ppm), low (0.10–0.22 ppm), and very low (< 0.10 ppm). The highest aquifer percentage in the low desert zone of the southwestern part of the study area is characterized by moderate, high, and very high concentrations of Pb. In contrast, the low and very low concentrations of Pb are concentrated in the eastern side of the investigated area, specifically the Nile floodplain aquifer system.

Cd map distribution was generated as shown in Fig. 9c. Cd values range from 0.0001 to 0.832 ppm, with a mean value of 0.207 ppm and a standard deviation of 0.194 (Table 4). It has been classified into five categories based on the natural break classifier: very high (> 0.36 ppm), high (0.23–0.36 ppm), moderate (0.14–0.23 ppm), low (0.06–0.14 ppm), and very low (< 0.06 ppm). The highest aquifer percentage in the low desert zone of the southwestern part of the study area is characterized by moderate, high, and very high concentrations of Cd. In contrast, the low and very low concentrations of Cd are concentrated in the eastern part of the study area, specifically the Nile floodplain aquifer system.

Cu map distribution was generated as shown in Fig. 9d. Cu values range from 0.008 to 1.001 ppm with a mean value of 0.421 ppm and a standard deviation of 0.333 (Table 4). It has been classified into five categories based on the natural break classifier: very high (> 0.62 ppm), high (0.47–0.62 ppm), moderate (0.33–0.47 ppm), low (0.16–0.33 ppm), and very low (< 0.16 ppm). The highest aquifer percentage in the low desert zone of the southwestern part of the study area is characterized by moderate, high, and very high concentrations of Cu. In contrast, the low and very low concentrations of Cu are concentrated in the eastern part of the study area, specifically the Nile floodplain aquifer system.

Based on the distribution maps of the heavy metals Zn, Pb, Cd, and Cu, it is evident that the high concentration of these elements is concentrated in the low desert zone aquifer system, with the concentration gradient decreasing towards the Nile Valley. Very high concentrations are observed in wells located very close to the sewage system, ponds, depressions, and wastewater basins. The very high zone is surrounded by high concentrations, which then transition to moderate, then low, and finally very low heavy metal concentrations. These data are confirmed by bacterial tests, where the very high and high zones are characterized by high bacterial contamination.

## Discussion

### Wastewater disposal site based on RS time series data

**Fig. 10:**
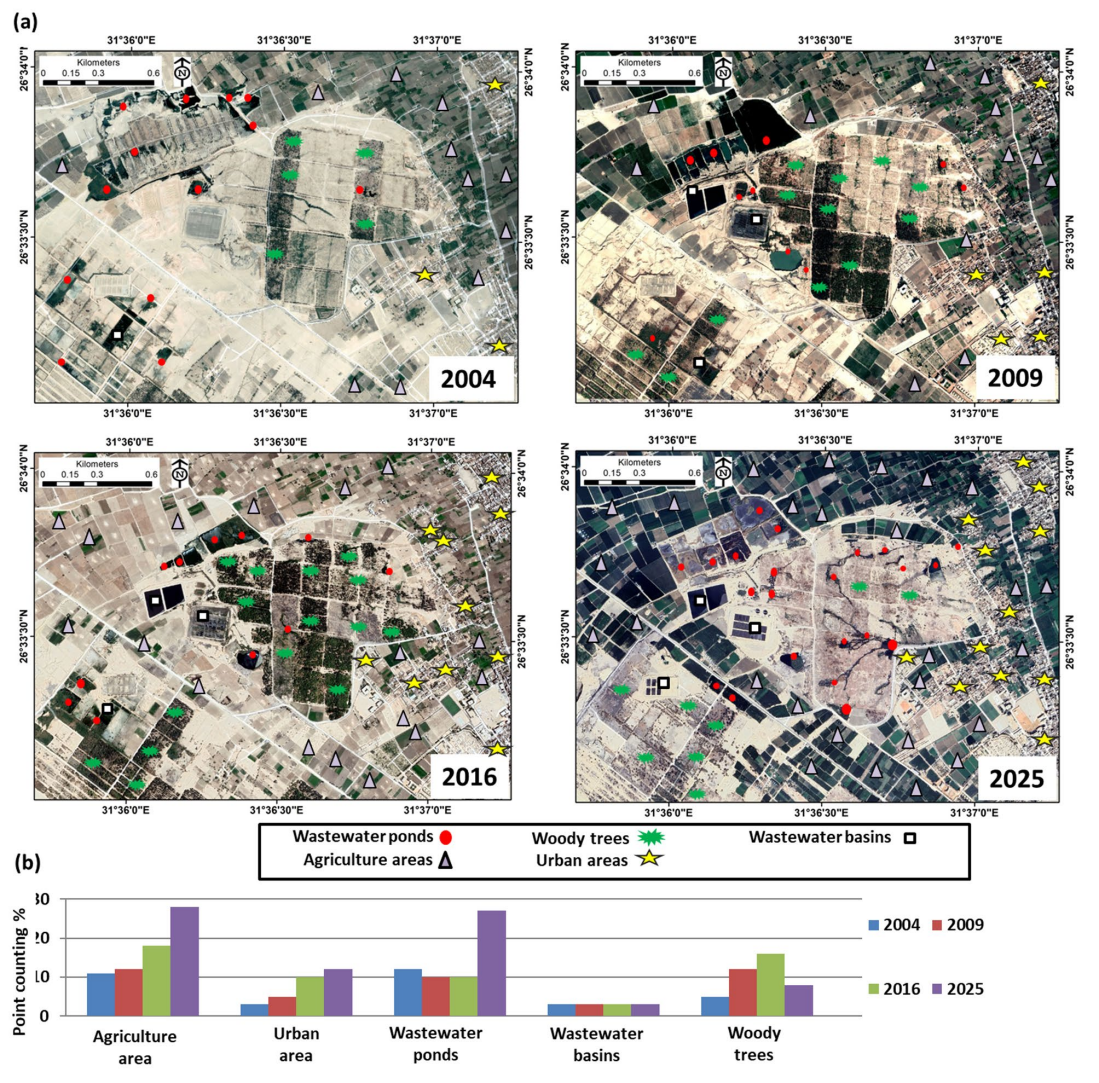
**a** A temporal sequence of high-resolution satellite images from 2004 to 2025 (from Google Earth Pro) illustrates the changes in waste disposal ponds, tree farms, and the rapid expansion of agricultural and urban areas in the west Sohag area. The 2025 image shows that the wastewater basins are in use, and some depressions are still filled with wastewater. (Source: the Google Earth professional images **a** were prepared by the authors (https://earth.google.com). **b** Histogram diagram showing the point counting percentage changes in each land use class for the years 2004, 2009, 2016, and 2025.

The study area is located in the desert zone, “the low desert zone,” west of Sohag Governorate, Egypt. The area covered by irrigated agriculture fields has increased dramatically since 2004. Figure 10 shows that between 2004 and 2025, the agricultural fields were mainly located around the wastewater disposal sites. The wastewater disposal ponds are located haphazardly in the west Sohag site. Later on, by 2018, the wastewater treatment station was expanded, and new wastewater basins were established; however, the ponds are still there, and water seeps downwards towards the groundwater aquifer. The wastewater disposal sites proved to have a negative impact on the environment. The bacteriological impacts on groundwater samples showed a positive indication of the presence of fecal coliform bacteria^38–40^. Remote sensing techniques and satellite images have proven highly effective in identifying hazardous zones caused by wastewater and landfill sites worldwide^18,32,41–43^. Our study utilized Google Earth Pro data to identify water ponds and generate a temporal sequence of high-resolution satellite images from 2004 to 2025 (Fig. 10a, b), aligning with other studies that employ Google Earth to detect water ponds using high-resolution images^44,45^.

Detailed monitoring of the changes in agricultural lands, urban areas, wastewater ponds, and a number of wastewater confined basins in the study area was carried out over the period (2004–2025). The results revealed highly hazardous environmental observations (Fig. 10a, b). The results showed that the agricultural areas increased by 60%, the urban areas increased by 75%, the wastewater ponds increased by 55% and the number of wastewater basins is constant. These results showed a rapid increase in the area of agriculture and urban areas, accompanied by a consistent rise in the number of wastewater ponds and a constant number of wastewater basins serving the study area. The continuous increase in agricultural and urban areas directly indicates an increase in population, which in turn exacerbates the environmental impact resulting from wastewater. A geographic information system GIS used the results of the geochemical analysis to produce spatial distribution maps for four important heavy metals in the groundwater aquifer west of the Sohag area: (a) Zn, (b) Pb, (c) Cd, and (d) Cu. This step aimed to detect variations in the concentrations of heavy metals in the groundwater to estimate the environmental effect of wastewater disposal sites on the groundwater aquifer in the study areas. The results proved the strong positive relationship between the wastewater sites and the concentrations of these heavy metals (Fig. 9).

### Scatter variation diagrams and factor analysis

#### Scatter variation analysis of heavy metals

**Fig. 11:**
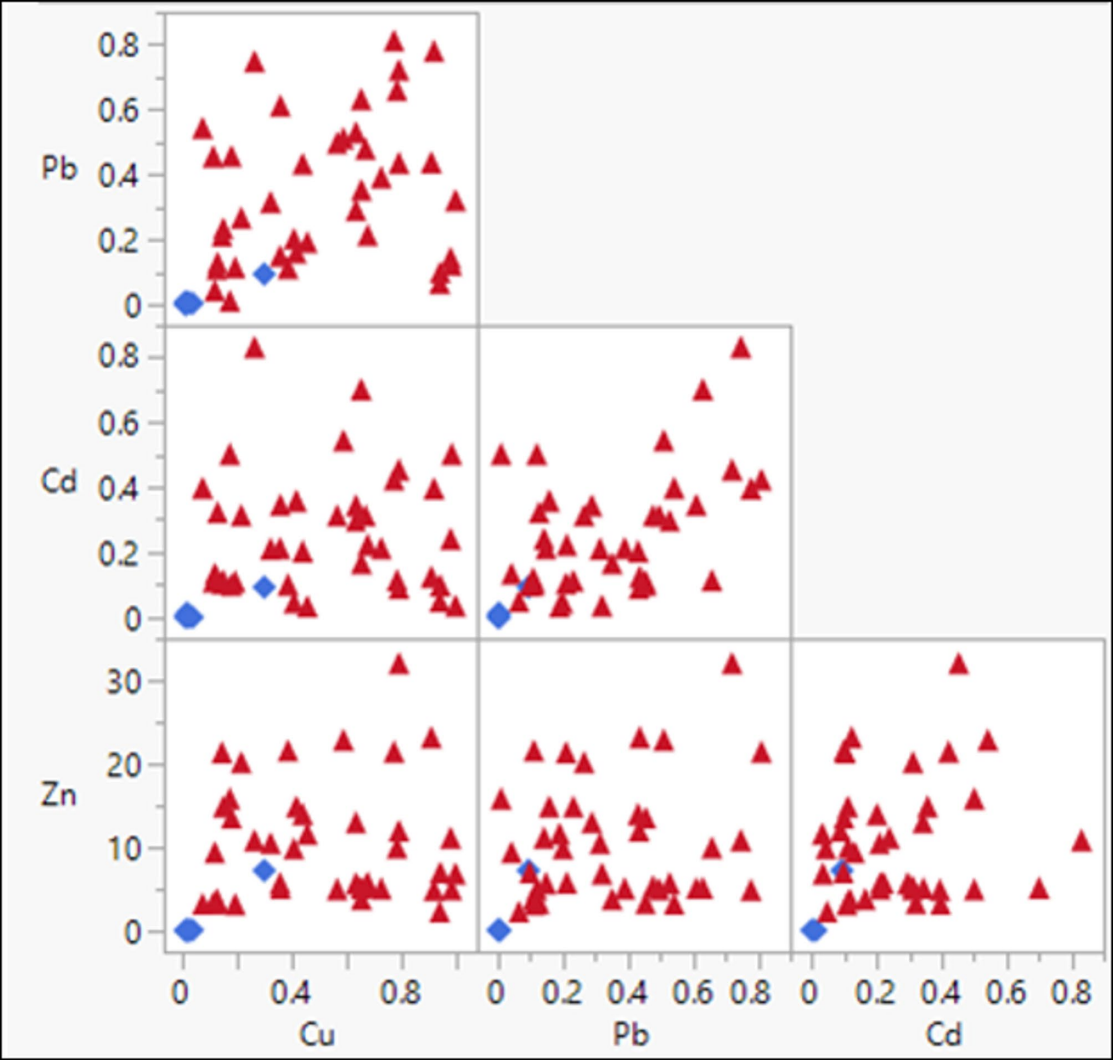
Scatter plot comparisons between data from the water wells in and surrounding the west Sohag disposal site and the data collected from the Nile floodplain.

The chemical analyses of the most serious elements (lead (Pb), Copper (Cu), Zinc (Zn), and cadmium (Cd)) have been evaluated based on the variation diagrams for all samples collected from the wastewater site, specifically the low desert zone aquifer (samples 1 to 40) and the Nile floodplain aquifer (samples 41 to 50) (Fig. 11). The results show a good correlation between the four heavy metals. Also, the concentration of heavy metals in west Sohag disposal site is higher than that in the surrounding data from the Nile floodplain, except for one sample, “sample number 46,” which is located in the floodplain aquifer and gives a higher heavy metal concentration than the floodplain samples. From the sample location, it was found that the water well is located along the boundary between the low desert zone and the Nile floodplain zone. The variation diagrams indicate that the aquifer under the low desert zone is contaminated with heavy metals.

#### Hierarchical cluster analysis (HCA)

**Fig. 12:**
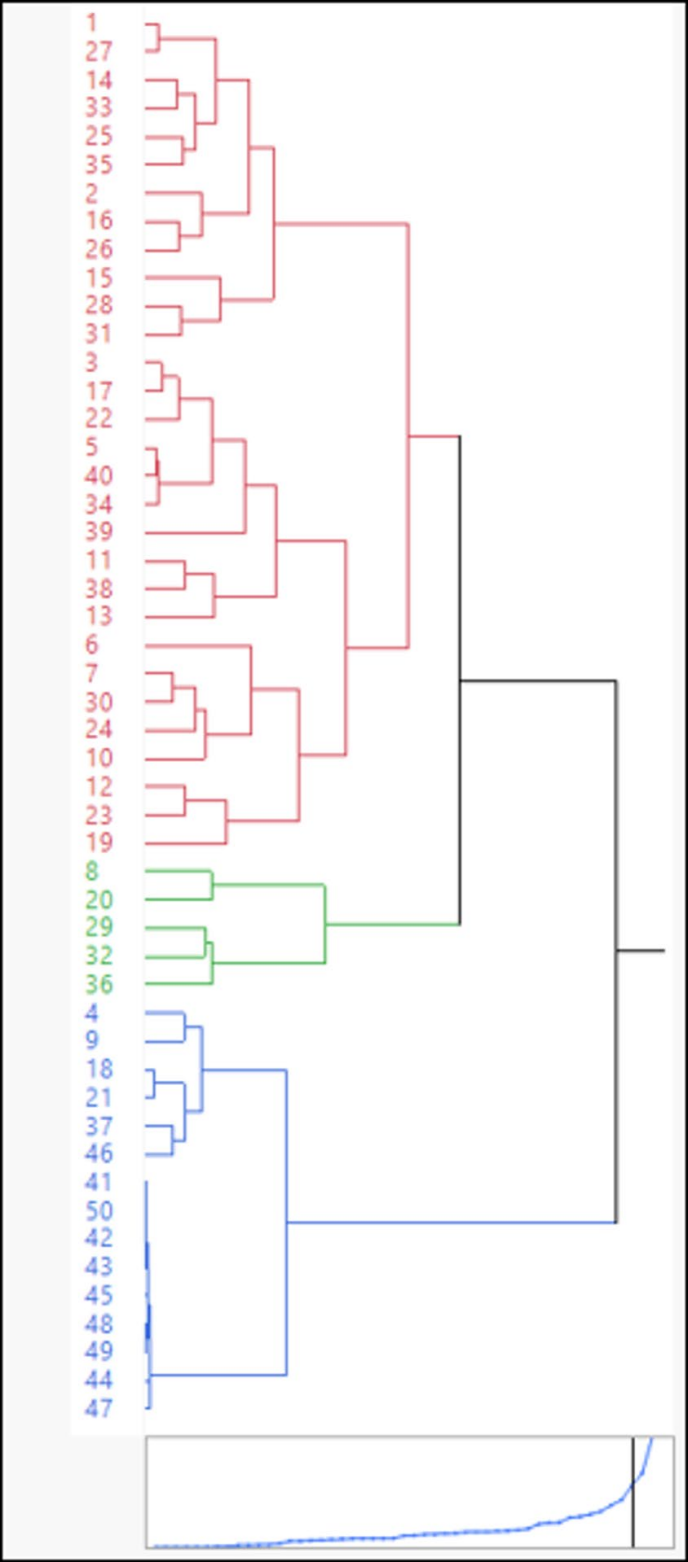
Hierarchical clustering plot for data comparisons between west Sohag samples from the disposal site and the Nile Valley floodplain samples.

HCA grouped the data by comparing the groundwater samples using heavy metals (Zn, Pb, Cd, and Cu), and the results were presented as a dendrogram (Fig. 12). The dendrogram results classified the groundwater types into three main groups, depending on the heavy metals. Group 1 contains samples with higher concentrations of Zn, Pb, Cd, and Cu, which are denoted by red colors. Within Group 1, three subgroups can also be distinguished. Group 2, with a green color, includes samples 8, 20, 29, 32, and 36. It can be assumed that Group 2 is a transition between Group 1 (high heavy metal concentration) and Group 3, with a blue color (low heavy metal concentration). Group 3 is characterized by its blue color and low heavy metal concentrations. In addition, group 3 will be subdivided into two subgroups. Data interpretation indicated that group 1 is high and very high contaminated zones, group 2 is the transition zone, and group 3 is a low and very low hazard zone. Pollutants are increasing in the groundwater aquifer west of the Sohag site area. This suggests that wastewater infiltration, which adds heavy metals to the aquifer system, is more effective in the west Sohag site, where the accumulation of these heavy metals in the groundwater is attributed to intensive human activities due to uncontrolled wastewater infiltration.

### Factor analysis

**Fig. 13:**
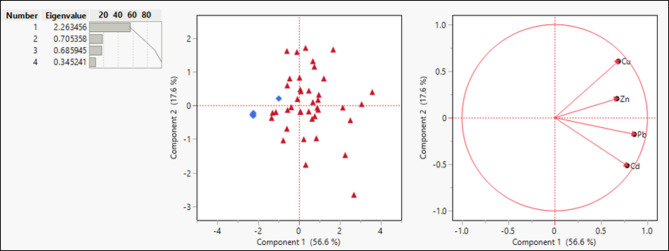
Factor analysis distribution curve **a** R-Mode and **b** Q-Mode.

Factor analyses were utilized to identify the factors that affect chemical elements fractionation. R and Q-mode factor analysis^46,47^ was achieved using the software JMP for the data (50 samples) (Fig. 13). The concentrations of the heavy metals (Zn, Pb, Cu, and Cd) were identified in the groundwater samples. These data were statistically graphed utilizing the factor analysis model (R and Q mode) to identify the interrelationship between these elements and samples from a certain source (Fig. 13a, b). It is possible to quantify the primary factors influencing the metals’ amounts and distribution. It is also possible to identify the anthropogenic and natural factors that affect metals’ behavior. The R-mode factor analysis was used to statistically quantify the inter-variable relationship and the potential factors influencing their behavior. The metals were separated into two geochemical trends by the loadings of the first and second components, which may indicate linked sources: (1) a wastewater group which includes the samples collected from the wells in the vicinity of the disposal sites (Red color), (2) another group that has very low concentration of heavy metals that are collected from the Nile flood plain aquifer (Blue color). In addition, factor analysis shows two sources of heavy metals: the first source is the heavy metals of Cu and Zn. The second source includes Pb and Cd. Generally, Pb- gasoline burning in automobiles is the main source of lead (Pb). In addition, lead (Pb) is utilized as a necessary production component in a number of industries such as painting, pipe/dye making, and battery industries. Copper (Cu) and Zinc (Zn) are also very harmful and toxic metals.

### Effect of heavy metal contamination on water quality

The natural sources of heavy metals in water are mainly related to the geology and hydrogeology of the aquifer. Our study indicated that the aquifer system is affected by high concentrations of heavy metals and decreases eastward towards the Nile flood plain. The study agrees with other studies dealing with heavy metals due to wastewater disposals. Rising population and overpopulation have affected groundwater quality, where one of the most detrimental compounds for aquatic pollution is heavy metal contamination^48^. The water quality is impacted by home and industrial pollutants^49^. Different pollutants, i.e., inorganic or organic compounds like pesticides with high concentrations of heavy metals, immediately affect water properties. The water-bearing formations in the study site have high horizontal and vertical permeability (horizontal permeability varies between 30 and 90 m/day), and specific storage varies between 6.7 × 10^−6^ and 7.6 × 10^−6^^48^. The thickness of water-bearing layers varies between 20 and 80 m and rises in the east direction (towards the Nile cultivated lands). The hydraulic conductivity varies between 0.29 and 3.72 m/day with an average porosity of 38.9%. These permeable layers can allow wastewater to rapidly infiltrate into the groundwater aquifer, posing a significant threat to the groundwater quality. These results are similar to other studies investigating the effects of wastewater ponds on other areas in Egypt^50,51^. These impacts include depletion of the groundwater table, and soil salinization is one aspect of the land degradation process. Still, some heavy metals are essential for metabolic action even in minute quantities. Nonessential heavy metals in high concentrations act as essential metals at a certain point and affect the main critical cellular functions, which frequently cause acute toxic bioaccumulation^52^. Anthropogenic activities are identified as the primary cause of heavy enrichments in aquatic environments^53^.

Based on the WHO standard values for the four heavy metals in the study area, Table 6. It was found that all samples have Copper values less than the maximum allowable limits, however, for Pb, Cd, and Zn seven samples have values less than the maximum allowable limits (samples 41, 42, 44, 45, 47, 49, and 50) which are located in the floodplain of the River Nile, whereas the rest of the samples that located in the vicinity of the wastewater sites have a values above the maximum allowable limits and increase as we moved west wards towards the contaminated sites.

**Table 6:** Heavy metal concentration comparisons with the WHO maximum allowable concentrations for Zn, Pb, Cu, Cd.

WHO values		Study area samples	
Heavy metal	Maximum allowable limits (mg/l)	Samples under the MAL	Samples above the MAL
Zn	0.2	41, 42, 44, 45, 47, 49, 50	1 to 40, 43, 46, 48
Pb	0.01	41, 42, 44, 45, 47, 49, 50	1 to 40, 43, 46, 48
Cu	2	1 to 50	No samples
Cd	0.003	41, 42, 44, 45, 47, 49, 50	1 to 40, 43, 46, 48

### Strategic management framework for contaminated areas

To overcome the wastewater problems and the contamination of the groundwater aquifer by heavy metals, a management framework can help local authorities take proper action as follows: (1) identify the source and prepare a risk map by the following: (a) determining the hydrochemical fingerprints that help trace the origin of contaminants (industrial, agricultural, anthropogenic, or natural geogenic); (b) building a GIS-based contamination map in which a visualization of the spatial distribution of various heavy metals is made, zoning regulations are determined, and the remediation methods are determined, and (c) preparing a vulnerability assessments in which identify the aquifer portions that at risk based on the permeability and closeness to the pollution sources. (2) Regulatory enforcement and wastewater controls, which involve (a) recalibrating the pollution permits and discharge limits in accordance with the updated pollution levels, (b) Mandatory effluent treatment for the wastewater, and (c) avoiding dumping wastewater in desert depressions without treatment. (3) Water quality monitoring in which (a) tracking the metal concentrations in groundwater wells and ponds, (b) activate the community and public engagement, and (c) apply a protocol for collecting seasonal samples to determine the contamination levels. (4) Apply a remediation strategy that includes (a) pump and treatment systems for localized contaminated wells, (b) establish a reactive barrier using activated carbon to intercept the contaminated plumes, and (c) establish a phytoremediation zone near the recharge areas.

## Conclusions

The proper assessment of an area’s suitability for wastewater disposal must take into account not just surface sediment qualities, groundwater aquifer conditions, and geological context, but also prospective and present land use. Most of the land features should be determined during the field survey. Consequently, general and detailed land use maps will be an essential part of future planning activities. Land use maps should be substantiated for the entire low desert zone and the new flood plain of the River Nile for the west Sohag site. This work presents the characterization of a wastewater disposal site using geotechnical, geochemical, and GIS land use techniques. The results of the site characterization and investigation for the proposed wastewater disposal sites, including geochemical analysis and hazard maps, indicate that some of these sites are unsuitable locations for the land application of wastewater.

The average heavy metal concentration of groundwater decreased in the order Zn > Cu > Pb > Cd, with mean values of 8.55, 0.421, 0.282, and 0.207 ppm, respectively. Hierarchical cluster analysis and factor analysis revealed that pollutants are increasing in the groundwater aquifer from the west of the Sohag site area. The results of this study, which utilize GIS and geochemical approaches to highlight potential issues, represent a significant advance for the Sohag district authorities and residents, as they focus attention on existing and future problems associated with wastewater treatment sites. Finally, it was found that in areas such as west Sohag, where agricultural intensity and urban expansion intersect, results showed elevated levels of heavy metals (e.g., from fertilizers and wastewater). To avoid the problem, two steps are essential: (1) apply public health safeguards, which include (a) finding an alternative water supply for the people in the contaminated area, (b) conducting a health screening for the people who are exposed to high levels of heavy metals, and (c) establishing educational workshops to teach people the safe water use and avoid contaminated water in ponds for agricultural and domestic activities. (2) Long-term planning that includes (a) land use zonation to avoid activities near aquifers, (b) constructing buffer zones around wastewater ponds, (c) non-use of untreated wastewater, and (d) establishing a coordination network between various agencies such as health, water, agriculture, and universities.

## Data Availability

The authors confirm that the data supporting the findings of this study are available within the article. The datasets generated during and/or analyzed during the current study are available from the corresponding author on reasonable request.
